# Nematicidal Potential of Sulla (*Hedysarum coronarium* L.) against the Root-Knot Nematode *Meloidogyne incognita*

**DOI:** 10.3390/plants11192550

**Published:** 2022-09-28

**Authors:** Trifone D’Addabbo, Aldo Tava, Maria Pia Argentieri, Elisa Biazzi, Vincenzo Candido, Pinarosa Avato

**Affiliations:** 1 CNR-Institute for Sustainable Plant Protection, 70125 Bari, Italy; 2 CREA-Research Centre for Animal Production and Aquaculture, 26900 Lodi, Italy; 3 Department of Pharmacy–Drug Sciences, University of Bari Aldo Moro, 70125 Bari, Italy; 4 Department of European and Mediterranean Cultures, University of Basilicata, 75100 Matera, Italy

**Keywords:** *Hedysarum coronarium*, flavonoids, condensed tannins, nematicidal, *Meloidogyne incognita*

## Abstract

The content of nematicidal metabolites such as saponins, flavonoids and tannins in sulla (*Hedysarum coronarium* L.) suggests its potential nematicidal activity. In this study, the biocidal activity of 62.5–1000 μg mL^−1^ concentrations of flavonoid and tannin fractions from sulla was assessed in in vitro assays on the infective juveniles (*J2*) of the root-knot nematode (RKN) *Meloidogyne incognita*, while the suppressive effects of soil amendments with 10–40 g kg^−1^ soil rates of sulla biomass were investigated on potted tomato infested by *M. incognita*. The content of total nitrogen, carbon, flavonoids, tannins and saponins of sulla experimental material was also determined. After a 96-h exposure, more than 80% of the *M. incognita J2* were killed even by a 125 µg mL^−1^ concentration of the flavonoid extract, while mortality peaked at 89% only at the 1000 µg mL^−1^ concentration of the tannin solution. Soil incorporation with sulla biomass significantly reduced the *M. incognita* densities both on tomato roots and in the soil, compared to either the non-treated control and chemical treatment with Fluopyram. The data confirmed the nematicidal potential of sulla, mainly due to its content of flavonoids and tannins, suggesting its suitability as green manure or a soil amendment for sustainable RKN management.

## 1. Introduction

The microscopical size and non-specific symptoms often lead to an underestimation of root-knot nematode (RKN) (*Meloidogyne* species) attacks, while it has been estimated that these phytoparasites are annually responsible of about 12–15% of world crop losses [1,2]. Increasing consumer environmental awareness and severe restrictions to the use of crop pesticides imposed by EU regulations are leading to a progressive substitution of synthetic nematicides with more sustainable alternatives such as plant-based agronomical techniques (green manures, crop rotations and others) and botanical products [3,4,5]. Studies of our research groups highlighted the nematicidal properties of specialized metabolites from a large variety of plant families, such as glucosinolates from Brassicaceae species [6], sesquiterpenes from Asteraceae [7], phenolics and alkamides from *Echinacea* species [8], or essential oils from aromatic and medicinal plants [9,10,11,12].

The literature data indicate that plants from the Leguminosae family can also represent a potential source of new nematicidal products, due to their content of bioactive compounds such as saponins, polyphenols and alkaloids [13,14,15]. In previous investigations, saponin fractions from various *Medicago* species were found to be strongly active on the infective juveniles (*J2*) of the RKN *Meloidogyne incognita* Kofoid et White (Chitw.), as well as against mixed stages of the virus-vector nematode *Xiphinema index* Thorne et Allen [14,16,17,18]. The field exploitation of the nematicidal potential of Leguminosae plants mainly relies on their agronomical use as green manures and soil amendments with dry biomasses. A number of previous experiments in soil documented a strong suppression of RKN infestation by soil amendments with biomass from *Medicago* species [14,17,19] or by green manures or crop rotations with Leguminosae plants from both temperate regions (*Trifolium* spp., *Vicia* spp., *Lupinus albus* L.) and tropical or sub-tropical areas (*Mucuna* spp., *Crotalaria* spp.) [20,21,22].

The Leguminosae family also includes the genus *Hedysarum* L., consisting of about 160 species widely distributed in the temperate regions of the northern hemisphere, and often reported for their pharmaceutical and biological properties [23]. Within this genus, *H. coronarium* L, commonly known as sulla or French honeysuckle, is a biennal plant native to the Mediterranean region, growing wild or cultivated for animal feeding [24]. According to the literature data, the chemical composition of *H. coronarium* is characterized by a high content of condensed tannins (proanthocyanidins), flavonoids and saponins [25,26]. All of these phytochemicals are known for a number of biological and pharmacological effects [13], among which is their nematicidal activity [17,27,28]. Soyasaponin I, i.e., the main saponin constituent of *H. coronarium* [25], was stated to have only a moderate biocidal activity on the grapevine nematode *X. index* [18]. Condensed tannins were reported for anthelmintic effects on gastrointestinal nematodes [29]; additionally, soil treatments with tannin-rich plant materials were documented for an effective control of RKN such as *M. javanica* Treub and *M. arenaria* Neal [30,31]. Moreover, pure flavonoid compounds, such as kaempferol, myricetin, quercetin and rutin, or extracts high in flavonoids were already documented as being toxic to *M. incognita J2* [32,33] and the model nematode *Caenorhabditis elegans* Maupas [34].

The presence of bioactive flavonoids and condensed tannins in sulla allows us to hypothesize a potential nematicidal activity of these chemicals and, therefore, a potential role of this plant in sustainable nematode management strategies. In the absence of previous literature data, the objective of this study was a first assessment of the toxicity of flavonoid and condensed tannins from sulla to the infective *J2* of *M. incognita,* along with the suppressiveness of soil amendments with sulla plant materials on *M. incognita* infestation on tomato (*Solanum lycopersicum* L.).

## 2. Results

### 2.1. Chemical Composition of Plant Biomass and Flavonoid and Tannin Extracts

The experimental green biomass of *H coronarium* was characterized for its C and N contents, and for the presence of the most dominant specialized metabolites, tannins, flavonoids and saponins (Table 1).

The total carbon and nitrogen content ranged 42 and 3% of the biomass dry weight, respectively. Chemical analysis revealed the presence of a high content of condensed tannins (119.68 ± 4.27 mg g^−1^ dw) and a low amount of phenolic compounds (4.89 ± 0.74 mg g^−1^ dw), which included both flavonoids (flavones and isoflavones) and simple phenolic acids (gallic acid, ferulic acid sinapic acid ecc.). The total saponins were quoted as 3.13 ± 0.23 mg g^−1^ dw, with soyasaponin I as the main component. The only two detected sapogenins were soyasapogenol B, representing the main compound (1.86 ± 0.25 mg g^−1^ dw), and soyasapogenol E (0.09 ± 0.03 mg g^−1^ dw).

**Figure 1:**
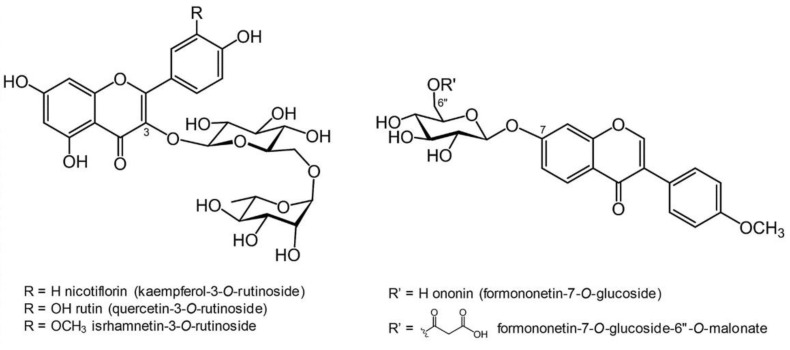
Chemical structure of the most abundant flavonoids detected in the *H. coronarium* extract.

The major constituents of the flavonoid extract were glycosyl derivatives of the flavonol kaempferol and quercetin, and the isoflavone formononetin (Figure 1).

Rutin (quercetin-3-*O*-rutinoside) was the most abundant compound (26.3% of the total extract), followed by nicotiflorin (kaempferol-3-*O*-rutinoside), which was detected as 13.3% of the total extract. Ononin (formononetin-7-*O*-glucoside) and its 6”-*O*-malonyl derivative were also detected in relatively high amounts, at 7.8% and 9.2%, respectively. A minor amount of isorhamnetin-3-*O*-rutinoside (4.8%) was also quantified in the extract.

The tannins were predominantly of the prodelphinidin type, dominated by *cis* extender units and *trans* terminal units. The prodelphinidin:procyanidin ratio was about 85:15, with a mean degree of polymerization of about 23 units. All of these results were in agreement with the data previously reported for *H. coronarium* collected in Southern Italy [25].

### 2.2. Toxicity of Flavonoids and Condensed Tannins to M. incognita Juveniles

The mortality of *M. incognita J2* was always nil or negligible, both in water and in the ethanol solution (Table 2).

The viability of *J2* was not affected by a 24-h exposure to almost any of the tested concentrations of the *H. coronarium* flavonoid fraction, as the nematode mortality ranged from 3.7 to 6.3% up to the 500 μg mL^−1^ concentration, and peaked at about 24% only with the 1000 µg mL^−1^ solution.

A similar mortality rate was reached at a much lower concentration, i.e., 125 µg mL^−1^, by prolonging the *J2* exposure for 48 h. At this exposure time, extract solutions ≥ 250 µg mL^−1^ were able to cause about 60–69% mortalities. A 96-h exposure of *J2* to *H. coronarium* flavonoid extract was strongly toxic at almost all of the concentrations, as more than 80% of the exposed specimens died at a 125 µg mL^−1^ concentrations, while the 500 and 1000 μg mL^−1^ solutions caused more than 90% mortality, which were also not significantly different from the same exposure to the Fluopyram solution.

The condensed tannin fraction of *H. coronarium* extract was poorly toxic to *M. incognita J2* at both 24- and 48-h exposures, as a negligible *J2* mortality was recorded at 62.5–500 µg mL^−1^ concentrations, while only the 1000 µg mL^−1^ tannin solution resulted in 15 and 38% mortality rates after 24- and 48-h exposures, respectively (Table 3).

A 96-h exposure remarkably increased the activity of tannin solutions on *M. incognita J2*, as about 58% mortality occurred at a 250 µg mL^−1^ concentrations, with a peak of 89% mortality in the 1000 µg mL^−1^ tannin solution. As in the experiment on the flavonoid extract, the *J2* viability was poorly affected by the ethanol solution, as well as being almost nil in the water control.

Values of LC_50_, i.e. the concentration causing a 50% mortality, were above the range of tested concentrations at the 24-h exposure to both flavonoid and tannin fractions, and for the 48-h treatment with tannin extract (Table 2). At the 96-h exposure, LC_50_ averaged 97 and 272 µg mL^−1^ for the flavonoid and tannin extracts, respectively, confirming the higher nematicidal activity of the flavonoid fraction.

### 2.3. Suppressiveness of Sulla Biomass to M. incognita Infestation

**Figure 2:**
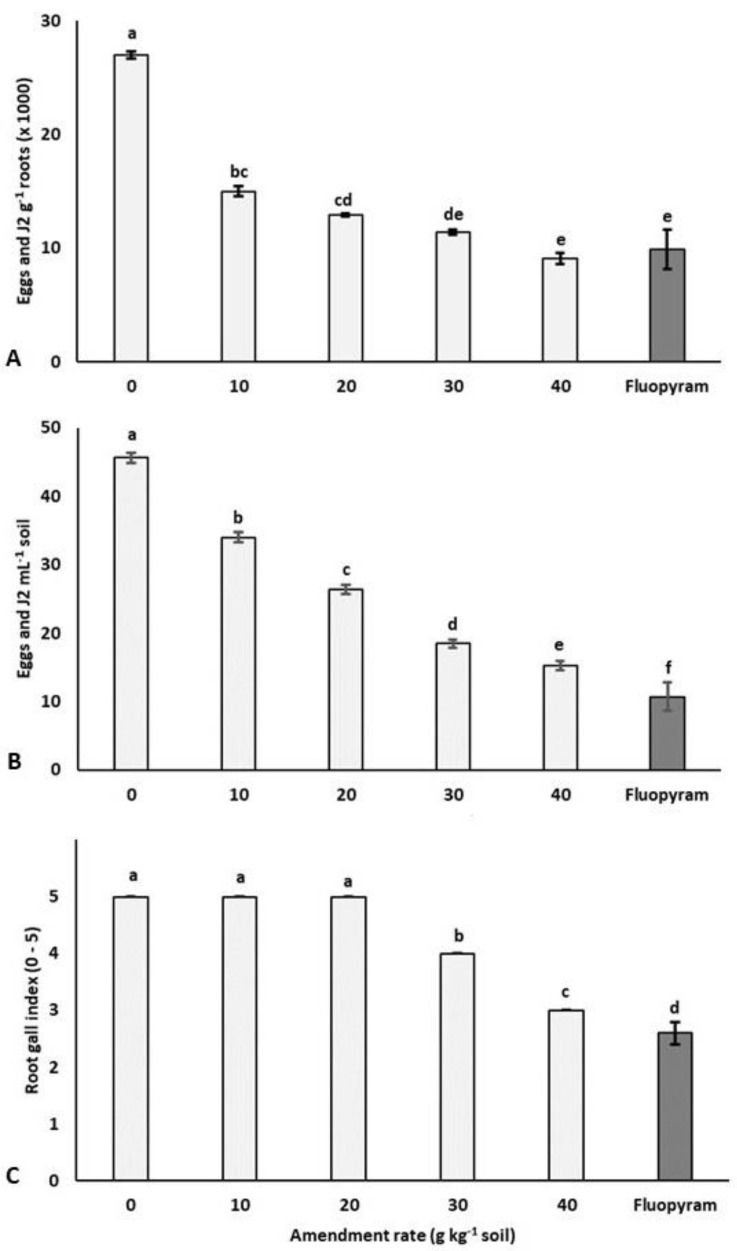
Effect of soil amendments with 10 – 40 g kg^−1^ soil rates of dry biomass of *Hedysarum coronarium* and Fluopyram (0.625 L ha^−1^) on the multiplication of *Melodogyne incognita* on tomato roots (**A**), in soil (**B**), and on root gall formation (**C**). The values are averages of five replicates. Bars marked with the same letter are not significantly different according to the LSD test (*P* < 0.05).

Soil amendments with all of the tested rates of sulla biomass significantly reduced both *M. incognita* multiplication on tomato roots (Figure 2A) and the final nematode population in the soil (Figure 2B).

Conversely, a significant reduction of gall formation on tomato roots occurred only at the two highest biomass dosages (Figure 2C). The number of nematode eggs and *J2* on tomato roots from soil amended with the 30 and 40 g kg^−1^ rates of sulla plant material was not significantly different from the treatment with the Fluopyram formulation, while the chemical treatment always resulted in a significantly lower nematode population in soil and root gall formation.

**Figure 3:**
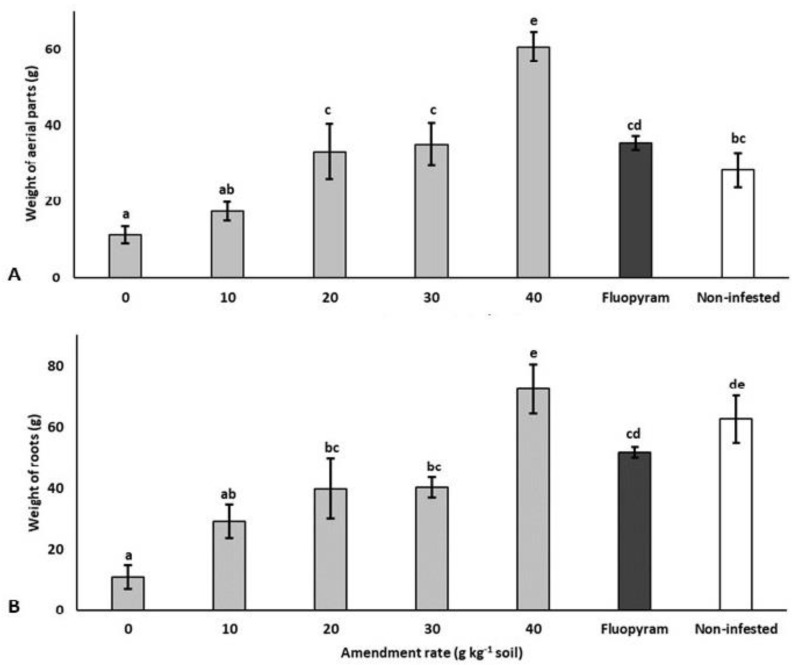
Effect of soil amendments with 10–40 g kg^−1^ soil rates of dry biomass of *Hedysarum coronarium* and Fluopyram (0.625 L ha^−1^) on the growth of the aerial parts (**A**) and roots (**B**) of tomato plants in soil infested by *Meloidogyne incognita*. The values are the averages of five replicates. Bars marked with the same letter are not significantly different according to the LSD test (*p* < 0.05).

Soil incorporation with sulla biomass also generally resulted in a significant increase of weight of both tomato aerial parts and roots of plants compared to the non-treated control and, at the highest rate, even to the treatment with Fluopyram (Figure 3). In addition, plants from soil amended with the 40 g kg^−1^ soil rate also showed no growth difference from the non-infested control.

## 3. Discussion

This study is the first report of the suppressiveness of sulla to RKN, though a similar activity was widely documented for other Leguminosae plants from temperate areas (*Trifolium* spp., *Vicia* spp., *Lupinus albus* L.) and tropical or subtropical leguminous species (*Mucuna* spp., *Crotalaria* spp.) applied as green manures or included in cultural rotations [20,21,22]. In previous studies, a strong suppression of *M. incognita* infestation on tomato was also achieved by soil amendments with powdered or pelleted dry biomass from different *Medicago* species [14,17]. 

A major role in RKN suppression by *Medicago* plants was played by their high content of bioactive saponins [14,17]. Conversely, the saponin fraction should reasonably give a poor or nil contribution to the suppressive performance of *H. coronarum* due to the poor nematicidal activity of its main saponin component, soyasaponin I [18]; therefore, it was not tested in the current study.

Data from the lab assays on *M. incognita J2* clearly indicated flavonoids and tannins as the main agents of the nematicidal properties of sulla but, according to the LC_50_ values at the 48- and 96-h exposures, the nematicidal activity of flavonoids was significantly higher compared to that of tannins. In this study, these compounds were tested separately, but further investigations should be carried out in order to assess the potential synergistic effects of their combination, as has previously emerged for other plant compounds [12].

Plant phenolics—as simple cinnamic acid derivatives like flavonoids and complex polyphenols such as tannins—are molecules which, due to their structural and steric features, display several biological properties, including antioxidant potential, radical scavenger activity, and metal chelating properties. In our previous studies, we have already observed a strong inhibition against *X. index* females by the simple phenolics, caffeic and chlorogenic acids [7], while other authors have studied the nematicidal activity of flavonoids or flavonoid-rich extracts against different nematode species [32]. The flavonols kaempferol, myricetin, quercetin and rutin were reported as the most lethal to *M. incognita J2*, mainly due to the activity of their oxidation-degradation products [32,35]. The high nematicidal activity of sulla flavonoids against *M. incognita J2* observed in this study is consistent with previous findings, with glycosyl derivatives of kaempferol and quercetin being the major flavonols in this plant material [25].

In this study, the condensed tannin extract from sulla showed a substantial inhibitory activity against *M. incognita J2*, though it was lower compared to that of the flavonoid extract. To the best of our knowledge, a nematotoxic activity of tannins against *M. incognita J2* was reported by only one previous study [36], though tannins from sulla were active against *M. incognita J2* at much lower concentrations and shorter exposure times (60% inhibition at 250 μg mL^−1^ after 96 h of incubation) compared to previous findings (45–70% after 10 days of exposure at rates of 0.30–1.50 g L^−1^). Tannins have been recognized for their nematicidal activity against several species of phytoparasites both in vitro and in soil experiments [37]. Thus, isolated condensed tannins from the rind of *Punica granatum* L. were able to inhibit the pinewood nematode *Bursaphelenchus xylophilus* Nickle, while hydrolyzable tannins from chestnut (*Castanea sativa* Mill.) were proven to have highly toxic effects against the egg hatching of RKN *M. javanica* [30]), and potato and carrot cyst nematodes *Globodera rostochiensis* Wollenweber and *Heterodera carotae* Jones, respectively [38,39]. Furthermore, soil treatments with condensed tannin extracts from *Schinopsis lorentzii* Engl. and *Acacia mollissima* Auct. were reported as being strongly active against *Longidorus elongatus* (de Man) Thorne et Swanger and *M. arenaria* [40]. Based on this experimental evidence, the European Food Safety Authority has recently approved the use of *Castanea* and *Schinopsis* tannins as soil nematicides for plant protection [41].

In agreement with data previously reported for the composition of *H. coronarium* collected in Southern Italy [25], the tannins identified in the plant material used in this study are condensed tannins of the prodelphinidin type, with catechin as the dominant unit, and gallocatechin and epigallocatechin as the dominant extender units. Due to the high number of hydroxyl groups in their structure, these specialized metabolites have a great ability to complex with proteins, and possibly to exert a defence strategy against pathogens [13,42,43,44].

In addition to the content of flavonoids and condensed tannins, the strong suppressiveness of sulla biomass to *M. incognita* observed in the experiment on tomato can be also contributed by the ammoniacal nitrogen generally released during the decomposition of legume crop green manures in soil [45]. Moreover, a further contribution to the reduction of RKN infestation can be attributed to the development of nematode-antagonistic microflora, as generally occurring on organic matrices such as the sulla biomass [46].

Tomato growth improvement following soil incorporation with sulla biomass is in agreement with the biostimulating effect observed after soil amendments with other leguminous plant biomasses [14,17]. Besides the positive effects of the reduced nematode infestation on plant fitness, tomato growth stimulation should also be attributed to the general improvement of soil physical, chemical and microbiological properties occurring after soil organic amendments [47], as well as to the possible synergistic effects of both of the two major chemical classes of phenolic constituents.

## 4. Materials and Methods

### 4.1. Plant Material

Green biomass from flowering wild plants of *H. coronarium* L. was collected at the beginning of May from uncultivated soil at Irsina (Matera, Basilicata region, Italy; 40° 47’ N, 16° 12’ E, 290 asl). The plant voucher specimen (HC101) was deposited at the Department of European and Mediterranean Cultures, University of Basilicata, Matera, Italy, and is available from the authors. The plant biomass was air-dried in the shade and then finely powdered, defatted with CHCl_3_ in a Soxhlet apparatus, and finally stored until its use for chemical analyses, the extraction of flavonoid and condensed tannin fractions, and soil amendments.

### 4.2. Nematodes

An Italian population of *M. incognita* from an infested tomato field at Castellaneta (Taranto, Apulia region, Italy) was identified by ITS region sequencing [48] and a sequence characterised amplified region (SCAR)-based PCR assay [49], and was multiplicated on tomato cv. Regina di Fasano in a glasshouse (25 ± 2 °C).

The *M. incognita J2* needed for the in vitro toxicity assays were obtained by incubating nematode egg masses, previously picked from the infested tomato roots, in distilled water at 25 °C in a growth chamber, and by storing the emerging *J2* at 5°C until use for the assays.

The nematode inoculum needed for the experiment in soil was prepared by finely mincing and thoroughly mixing the infested tomato roots and then shaking four random 10-g root samples for 3 min in a 1% NaClO aqueous solution [50]. The extracted eggs and *J2* were counted under a microscope, in order to assess the nematode density per gram of root.

### 4.3. Chemical Analyses

The green biomass was characterised for its content of total nitrogen, carbon, phenolics, tannins and saponins. The total nitrogen and carbon were measured according to the Dumas method [51], whereas the total phenolics content was determined by the Folin–Ciocalteu colorimetric method [52]. The content of condensed tannins was assessed by the butanol/HCl method [53]. The amount of saponins was determined as reported in Tava et al. [54]. Saponins were also characterised by evaluating their aglycone composition by the gas chromatography (GC) and GC/mass spectrometry (MS) of derivatised sapogenins, as obtained after acid hydrolysis [55]. Flavonoids and condensed tannins were characterized by HPLC/DAD, LC-ESI-MS analysis and NMR, as reported by Tava et al. [25].

### 4.4. Extraction and Purification of the Flavonoids

Defatted plant material (250 mg) was extracted with 750 mL of 80% MeOH under stirring overnight. After centrifugation at 3000× *g* for 10 min, the supernatant was separated, and the residue was re-extracted with 750 mL 80% MeOH. The combined extracts were diluted to 30% MeOH and applied onto a RP18 column (100 × 60 mm, 40–63 μm, Merck, Milano, Italy) preconditioned with 30% MeOH. The column was washed with 30% MeOH (500 mL) to remove soluble compounds, and then with 80% MeOH (500 mL) to elute flavonoids. The methanolic solution was evaporated to dryness under reduced pressure at 40 °C in order to obtain 8.2 g crude flavonoid extract. The extract was dissolved in 2% ethanol and used in the experiments. The flavonoid extract was characterized by HPLC/DAD and LC/MS, and mass spectra were acquired both in positive and negative ion mode. The identification of the components of the extract was performed comparing their retention times, UV absorptions and mass spectral data with those of the available standard compounds, as well as with previously identified constituents of *Hedysarum* spp. and other data reported in the literature [25,26,56].

### 4.5. Extraction and Purification of Condensed Tannins

Dried leaves of sulla (250 g) were extracted with 750 mL acetone/water (7:3 *v*/*v*) under stirring for 30 min. The extract was filtered using a paper filter, and the plant material was extracted again in the same conditions. The combined extracts were then concentrated in vacuo at 40 °C to remove acetone, and the obtained aqueous solution was defatted with CH_2_Cl_2_ (3 × 200 mL). From the aqueous layer, the residual CH_2_Cl_2_ was eliminated under vacuum, and then the solution was freeze-dried to yield 15.2 g of brown solid of crude condensed tannins. The extract was dissolved in 2% ethanol and used at different concentrations in all of the experiments.

### 4.6. In Vitro Assays on M. incognita J2

A 500−μL volume of a nematode water suspension, containing about 100 *M. incognita J2*, was pipetted in 1.5 mL Eppendorf vials and then added with the same volume of 125, 250, 500, 1000 and 2000 μg mL^−1^ concentrations of sulla flavonoid or tannin fractions in 2% ethanol water solutions, so as to reach final test concentrations of 62.5, 125, 250, 500 and 1000 μg mL^−1^. Distilled water, 2% ethanol solution and 0.625 μg L^−1^ water solution of a formulation of nematicide Fluopyram were included as controls. Nematode *J2* was incubated in a growth cabinet at 25 °C for 24, 48, or 96 h, providing four replicates of each concentration x exposure time combination. At the end of each exposure time, the *J2* from each replicate was checked under a microscope and recovered on a 5 μm sieve, repeatedly washed with water, and then transferred to distilled water for a further 72 h. The persistence of *J2* immobility in the following microscopical observation was assumed as evidence of their death. The nematode mortality rates of each treatment were calculated by Abbott’s formula, m = 100 × (1− nt/nc), in which m = percent mortality, nt = the number of viable nematodes after the treatment, and nc = the number of viable nematodes in the water [57]. The LC_50_ values of both the flavonoid and tannin fractions were calculated by the probit analysis of the mean mortality data. The experiments were repeated twice, and data from the two experimental runs were pooled in the absence of any significant experiment x treatment interaction.

### 4.7. Experiments in Soil 

A steam-sterilized (100 °C × 7 h) sandy soil (64.4% sand, 18.7% silt, 16.9% clay, 0.8% organic matter and 7.5 pH) was infested with the root inoculum of *M. incognita* described above, so as to reach an initial nematode density of about 15 eggs and *J2* mL^−1^ soil. The infested soil was then added with 10, 20 or 40 g kg^−1^ soil rates of the dry sulla biomass and poured into 1.5 L clay pots. The controls were represented by non-treated soil, either non-infested or infested with *M. incognita*, and by soil treated with the commercial formulation of Fluopyram, applied at a dose corresponding to a 0.625 L ha^−1^ field rate three days after transplanting and two weeks later. Five replicates of each treatment and control were provided, arranging the pots in a randomized block design on the benches of a glasshouse. A 1-month-old tomato (cv. Regina di Fasano) seedling was transplanted into each pot three weeks after the soil addition with sulla biomass and tomato plants.

The tomato plants were maintained at a constant 25 ± 2 °C temperature for two-months. At the end of this period, the plants were uprooted and the fresh weight of the aerial parts and roots was recorded for each plant. The gall formation on the tomato roots was estimated according to the 0–5 scale of Taylor and Sasser [58]. The number of eggs and *J2* per gram of root was assessed by processing a 10 g root sample from each tomato plant by the above-described Hussey and Barker method, while the nematode density in the soil was determined on a 500-mL sample from each by the centrifugal flotation method [59].

### 4.8. Statistical Analysis 

The data were arcsin-transformed for homogenizing error variances and then subjected to one-way ANOVA, followed by a comparison of the treatment means with Fisher’s Least Significant Difference pairwise procedure (*p* ≤ 0.05). All of the statistical analyses were performed using the software PlotIT 3.2 (Scientific Programming Enterprises, Haslett, MI, USA).

## 5. Conclusions

The data presented in this study clearly indicate the strong suppressiveness to *M. incognita* of plant materials from sulla, suggesting this species as an additional tool for sustainable nematode management strategies. Use as green manure seems to be the most suitable for the exploitation of the nematicidal potential of this plant, also in consideration of the agronomical benefits related to this practice. However, high attention should be given to seasonal period of green manures, so as to avoid spring–summer periods which are favourable to a potential RKN multiplication on sulla roots [60].

As also emerged from this study, an alternative agronomical exploitation could be represented by soil amendments with the dried biomasses of sulla crops from the same farm and specifically addressed to this use. A further alternative could be a granular or powder formulation of dry biomasses to apply directly for soil treatments, as corroborated by the presence on the market of similar derivatives of other nematicidal plants (biofumigating brassicaceous species, neem). The production of industrial formulates of extracts seems to be less feasible, as they are expensive and economically not competitive with the synthetic nematicides currently present on the market.

A careful evaluation of sulla agronomical techniques aimed to maximize the content of bioactive components, mainly flavonoids and tannins, as well as of the effects on beneficial soil biota, should be carried out preliminary to a potential exploitation of this plant as a nematode suppressant.

## Figures and Tables

**Table 1:** Composition of the experimental biomass of *Hedysarum coronarium*.

Parameter	Unit	Mean Value ± SE ^1^
Total C	(% dw)	41.66 ± 0.01
Total N	(% dw)	2.97 ± 0.03
Total tannins	(mg g^−1^ dw)	119.68 ± 4.27
Total phenolics ^2^	(mg g^−1^ dw)	4.89 ± 0.74
Total saponins	(mg g^−1^ dw)	3.13 ± 0.23

^1^ standard error. ^2^ Total phenolics include both flavonoids (flavones and isoflavones) and simple phenolic acids (gallic acid, ferulic acid, sinapic acid, ecc.), expressed as gallic acid equivalents.

**Table 2:** Percentage mortality (mean ± SE) of infective juveniles of *Meloidogyne incognita* after a 24-, 48-, or 96-h exposure to 62.5–1000 μg mL^−1^ concentrations of the flavonoid extract from *Hedysarum coronarium* biomass.

Concentration (μg mL^−1^)	Exposure Time (Hours)					
	24		48		96	
62.5	3.7 ± 0.7	b	4.7 ± 0.7	a	12.8 ±1.2	b
125	4.7 ± 0.7	bc	25.4 ± 0.8	b	80.3 ± 6.8	c
250	4.7 ± 0.3	bc	59.7 ± 2.0	c	89.6 ± 4.2	d
500	6.1 ± 1.1	c	65.4 ± 1.8	d	91.6 ± 2.1	d
1000	24.2 ±1.5	d	68.8 ± 0.5	de	93.2 ± 1.2	d
LC_50_	≫ *		350		97.2	
Fluopyram **	81.5 ± 1.8	e	80.7 ± 4.2	f	87.9 ± 2	cd
Ethanol (2%)	0.5 ± 0.3	a	0.6 ± 0.5	a	1.8 ± 0.6	a
Water	0.5 ± 0.3	a	0.5 ± 0.3	a	0.6 ± 0.6	a

* Values above the range of tested concentrations. ** 0.625 μg L^−1^. Means followed by the same letters on the same column are not significantly different (*p* ≤ 0.05) according to the Least Significant Difference Test.

**Table 3:** Percentage mortality (mean ± SE) of infective juveniles of *Meloidogyne incognita* after a 24-, 48-, or 96-h exposure to 62.5–1000 μg mL^−1^ concentrations of the tannin extract from *Hedysarum coronarium* biomass.

Concentration (μg mL^−1^)	Exposure Time (Hours)					
	24		48		96	
62.5	0.0	a	2.2 ± 0.2	ab	7.0 ± 1.4	b
125	0.0	a	2.7 ± 0.6	ab	22.3 ± 0.8	c
250	0.5 ± 0.5	a	5.6 ± 1.6	b	57.8 ± 2.7	d
500	1.7 ± 1.3	a	6.1 ± 2.9	b	71.0 ± 2.7	e
1000	15.0 ± 0.5	b	38.1 ± 1.5	c	88.8 ± 1.1	f
LC_50_	≫ *		≫*		272	
Fluopyram **	84.9 ± 2.8	c	86.9 ± 1.4	d	92.0 ± 0.8	f
Ethanol (2%)	1.1 ± 0.6	a	1.1 ± 0.7	a	3.0 ± 0.3	a
Water	0.3 ± 0.1	a	0.7 ± 0.5	a	0.7 ± 0.5	a

* Values above the range of tested concentrations. ** 0.625 μg L^−1^. Means followed by the same letters on the same column are not significantly different (*p* ≤ 0.05) according to the Least Significant Difference Test.

## Data Availability

All data supporting reported results are available from the corresponding author.
